# Investigation of *Wolbachia spp*. and *Spiroplasma* spp. in *Phlebotomus* species by molecular methods

**DOI:** 10.1038/s41598-018-29031-3

**Published:** 2018-07-13

**Authors:** Bilge Karatepe, Serap Aksoy, Mustafa Karatepe

**Affiliations:** 1 Niğde Ömer Halisdemir University, Bor Vocational School, Bor-Niğde, Turkey; 2 Yale University, School of Public Health, Department of Epidemiology of Microbial Diseases, New Haven, Connecticut USA

## Abstract

The aim of this study was to determine the presence of *Wolbachia* spp. and *Spiroplasma* spp. in natural populations of sand flies in Turkey by molecular methods. A total of 40 *Phlebotomus* specimens (19 female and 21 male) were used in this study. Genomic DNA from whole sand flies was isolated and *Wolbachia* spp. infection prevalence was investigated by using *Wolbachia* gene specific primer sets (*wsp* and *GroEL)*. In addition, the DNA were analyzed for the presence of *Spiroplasma* infections utilizing bacterium specific 16 S rDNA PCR-amplification primers. Results of this analysis showed a *Wolbachia* infection prevalence of 70% (28/40). There was no sex-bias in infection prevalence, being 76% (16/21) and 63% (12/19) in males and females, respectively. Analysis of *Spiroplasma* infections indicated that 26% (5/19) of female sand flies were positive for infection, while none of the screened males (0/21) were positive. Of the 40 sand fly samples, only 2 were found to be positive for both *Wolbachia spp*. and *Spiroplasma spp*. The present study demonstrates the presence of *Wolbachia* and *Spiroplasma* infections in the natural sand fly populations in Turkey. This is the first report on *Spiroplasma* infection in the sand flies from Turkey.

## Introduction

Most insect taxa have heritable endosymbiotic bacteria that are able to manipulate various physiological functions including the reproductive biology of their hosts. Several recent studies have shown that endosymbionts promote the resistance of insects to certain natural enemies, such as viruses, bacteria, fungi, nematodes and parasitic wasps. *Wolbachia* and *Spiroplasma* are the two most widespread and widely studied endosymbionts in insects^1–3^. Sand flies (Diptera, Psychodidae) are vectors of viral, bacterial and protozoal pathogens to humans and other animals. *Phlebotomus* spp. are the predominant vectors of the protozoan disease, leishmaniasis, both in the Old and New World^4^.

*Wolbachia* is an intracellular bacterial endosymbiont (Proteobacteria; Rickettsiales) that is found in various arthropod tissues (most commonly in the reproductive tissues) as well as in filarial nematodes^5,6^. It is estimated that 76% of the insect species are infected with *Wolbachia*^7^. *Wolbachia* is inherited by vertical transmission mechanisms and can result in reproductive pathologies in their host insects, included cytoplasmic incompatibility (CI), male killing, feminization and induction of parthenogenesis. Recent works show that *Wolbachia* could be utilized for biological control strategies in various insect species^8,9^.

Similar to *Wolbachia*, *Spiroplasma* endosymbiont is also a maternally transmitted bacterium that belongs to a genus of wall-less, motile, helical, Gram-positive bacteria. *Spiroplasma* are mainly found extracellularly in the hemolymph of their host insects and in some cases cause a male killing pathology^2,3^. Recent studies have shown that *Spiroplasma* are present in over 4–7% of all insect species^10^. It was also recently shown that *Spiroplasma* can protect its insect hosts from infections with pathogenic organisms and therefore is a potentially useful tool for the control of vector borne diseases^11,12^.

The aim of this study was to determine the presence and prevalence of *Wolbachia* spp. and *Spiroplasma* spp. among wild-caught sand flies in Antalya province of Turkey by using molecular methods. We discuss approaches based on heritable reproductive endosymbionts as new potential tools for vector control in endemic areas.

## Results

Detection of *Wolbachia* was accomplished using the *Wolbachia wsp* and *groEl* specific primer sets. The results of this analysis show that 70% (28/40) of sand flies tested were positive for both *Wolbachia wsp* and *Wolbachia groEl* PCR. Of the *Wolbachia* positive individuals, 76% (16/21) were male and 63% of (12/19) were female sand flies.

Analysis for the presence of *Spiroplasma* using 16 S rDNA gene specific primers indicates that 26% (5/19) of female sand flies were positive for infection, while none of the male sand flies sampled (0/21) were positive. Of the 40 sand fly samples analyzed, only two were found to be positive (5%) for both *Wolbachia* spp. and *Spiroplasma* spp.

The amplified products were sequenced and the DNA sequence was analyzed by BLASTN analysis. The BLASTN results indicate that the sand fly *Wolbachia wsp* sequence shares the highest identity with the partial surface protein gene sequences (*wsp*) of *Wolbachia* endosymbiont of *Bactrocera neohumeralis* (100% identity: GenBank Accession Number KC668323) as well as the mosquito *Armigeres subalbatus* (100% identity: GenBank Accession Number KY523672). However, given the high conservation of this sequence, many different *Wolbachia* isolates showed 100% identity over the region analyzed. However, it appears that this strain of *Wolbachia* belongs to the Supergroup A. The sequences of three clones derived from 3 infected sand flies were identical.

The sequencing of the *groEl* gene of the clones was identical to the partial sequence derived from a *Wolbachia* endosymbiont of *Drosophila melanogaster* (100% identity: GenBank Accession Number AE017196) and *D. simulans* wHa (100% identity: GenBank Accession Number CP003884) based on BLASTN analysis. All clones had identical sequence. Given the high conservation of the *groEL* gene sequences, the sandfly *Wolbachia* sequence showed 100% identity with many other insect *Wolbachia* symbionts. However, many of the characterized *Wolbachia* strains that showed high similarity belonged to again Supergroup A.

With regards to *Spiroplasma* PCR-amplification products, the fragments were sequenced and the DNA sequence was subjected to BLAST analysis. The closest resulting match of the putative 16 S rDNA fragment was to the 16 S rRNA gene from an uncultured *Spiroplasma* sp. clone A9-24 (100% coverage, 100% identity: GenBank Accession Number KT983889.1).

Obtained sequence data were deposited to GenBank under the accession numbers as follows: MH393924 for wsp *Wolbachia Phlebotomus*, MH393925 for groEL *Wolbachia Phlebotomus*, and MH393926 for 16 S *Spiroplasma Phlebotomus*.

The high similarities of these gene products to many of the related sequences in the database make detailed phylogenetic analysis difficult and will require sequence analysis of additional genomic loci with higher levels of sequence variability in these bacteria.

## Discussion

In the present study, we tested for the presence of *Wolbachia* in 40 sand flies using two independent genes, *wsp* and *groEl*. Our results showed that twenty-eight out of the 40 sand flies tested were positive for *Wolbachia*. In addition, 5 out of the 40 sand flies tested were positive for *Spiroplasma* based on *Spiroplasma* bacterium specific 16 S rDNA amplification. Interestingly, *Spiroplasma* infections were only identified in female sand flies suggesting a potential sex specific infection bias. Of the 40 sand fly samples tested, only two were found to be positive for both *Wolbachia spp*. and *Spiroplasma spp*.

In previous studies using similar PCR-based molecular methods, the prevalence of *Wolbachia* in both Old and New world sand fly species has been investigated. Ono *et al*.^13^ has reported that 27% of sand flies (*Lutzomyia* spp. and *Phlebotomus* spp.) were infected with *Wolbachia*. Benlarbi and Ready^14^ determined that in *Phlebotomus perniciosus*, 60.3% were positive for the B group wPrn strain of *Wolbachia* and in *P. papatasi*, 81.7% were positive for the A group wPap strain of *Wolbachia*. The A-group strain of *Wolbachia* (wPap) was also found in *P. papatasi* by PCR amplification by Parvizi *et al*.^15^. Matsumoto *et al*.^16^ found *Wolbachia* sp. in pooled sand fly samples (*P. perniciosus*, *P. mascittii*, *P. ariasi*, *Phlebotomus* sp., *Sergentomyia minuta*) in Marseille, France. The presence of *Wolbachia* was detected in three of 20 sand fly species, including *Lutzomyia trapidoi*, in which it frequently co-occurred with *Leishmania* parasites^17^. Parvizi *et al*.^18,19^ determined presence of *Wolbachia pipientis* in *P. perfiliewi transcaucasicus*, *P. mongolensis* and *P. caucasicus*. Bordbar *et al*.^20^ found that 65.5% of Iranian sand flies were positive for *Wolbachia* based on *wsp* gene amplifications. Vivero *et al*.^21^ detected *Wolbachia* in *Lutzomyia* species in Colombian Caribbean coast. Da Rocha *et al*.^22^ were found that *Wolbachia pipientis* was positive 2.5% in *Lutzomyia* species. Li *et al*.^23^ determined *Wolbachia* (8.6%) and *Spiroplasma* (2.3%) in *Phlebotomus chinensis* in Henan and Sichuan collections from China, respectively.

In the present study, the prevalence of *Wolbachia spp*. was found to be 70% in sand flies analyzed from Antalya province of Turkey along the Mediterranean. The rate of infection prevalence found in this study is similar to the results reported in Iranian sand flies by Benlarbi and Ready^14^ and Bordbar *et al*.^20^. Observed differences in prevalence of *Wolbachia* spp. may be associated with variation in the geographical locations and the species of sand flies sampled^13^.

Work by Aksoy and Rio^24^ and Rio *et al*.^25^ describe the potential role of symbionts, including *Wolbachia*, as a novel tool for control of parasitic diseases. *Wolbachia* is responsible for inducing a number of reproductive modifications that enable its spread and maintenance in natural insect populations. The most common effect is cytoplasmic incompatibility^8,9,13^. In one study, it has been shown that a natural *Wolbachia* infection in sand flies causes cytoplasmic incompatibility, leading to embryonic mortality in them^18,20^. *Wolbachia* also causes induction of parthenogenesis in female artropods, thus insect populations can be negatively impacted upon infection with *Wolbachia*^8,18^. In addition, introduction of new *Wolbachia* infections into *Drosophila* as well as mosquitoes have been shown to induce the host immune system to make them resistant to pathogenic viruses and parasites^26–28^. For these reasons, it has been suggested that *Wolbachia* infections can prevent the transmission of parasites in infected insects. If *Wolbachia* infections in sand flies also confirm increased resistance to *Leishmania* parasites or to viruses, it can be desirable to incorporate this endosymbiont as a biocontrol agent against sand fly-transmitted diseases.

Much of the research on *Spiroplasma* has focused on *Drosophila* species. *Drosophila melanogaster* one of the most commonly used model organisms, naturally harbours Spiroplasma^2,3,29–31^. The prevalence of male killing *Spiroplasma* was found to vary between 0% and 17.7% in different *Drosophila* species^31^. In this study we determined *Spiroplasma* positivity rate as 26% in female sand flies analyzed from the southern coast of Turkey. None of the male sand flies were positive for *Spiroplasma*. This observation suggests that in sand flies *Spiroplasma* may employ a male killing drive strategy although additional prevalence and functional data are required to support this hypothesis.

The present study was performed to investigate the presence or absence of reproductive associated endosymbiotic bacteria in sand flies in Turkey. Molecular studies in Turkey have previously reported that *Wolbachia* infection was detected in *Trissolcus* species, *Culex pipiens* and *Phlebotomus* spp^32–34^. Further studies are needed to determine the extent and the potential biological significance of *Wolbachia spp*. and *Spiroplasma spp*. infections in sand flies in Turkey.

## Materials and Methods

**Figure 1:**
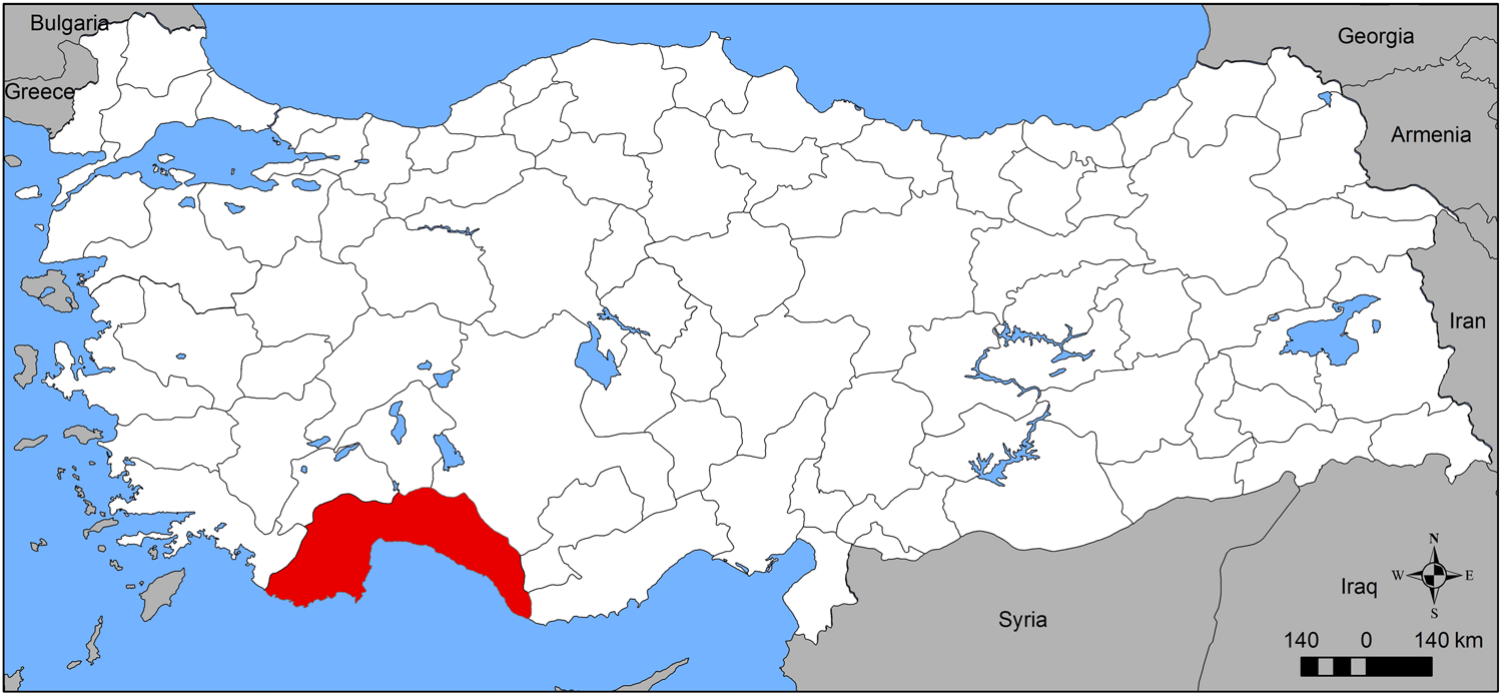
Location of Antalya province in Turkey (red colored area). Map shown in this article was created using ArcGIS 10.2 software by Esri.

This project was carried out to investigate the prevalence of *Wolbachia* spp. and *Spiroplasma* spp. in *Phlebotomus* flies by using molecular methods. A total of 40 *Phlebotomus* individuals (19 females and 21 males) were used in this study. Sand flies were collected from Antalya, in south of Turkey (with an altitude of 20 m, 36° 13′–36° 34′ N latitudes and 32° 15′–32° 38′ E longitudes), which has general characteristics of Mediterranean climate in August 2014 (Fig. 1). Sand flies were caught and identified with the help of the relevant resources^33,35^. Genomic DNA was extracted from whole sand flies using the DNeasy Blood and Tissue Kit (Qiagen, Valencia CA) according to the manufacturer’s instructions. The concentration of the extracted DNA was detected using a Nanodrop 2000 spectrophotometer and keep at −20 °C for further molecular experiments.

Genomic DNA integrity was tested by standard Polymerase Chain Reactions (PCR) amplification using primers against sand fly beta-tubulin (forward primer 5′ GCC TCC TGG TAT TGT TGG T 3′, reverse primer 5′ CTC ACG CAG CAG ATG TTC 3′, product size 490 bp). PCRs were performed in a total volume of 25 µl using 1 µl of DNA template and GoTaq Green Master Mix (Promega, Madison, WI, USA) following the manufacturer’s instructions. Thermocycling conditions were as follows: 94°C for 3 minutes; 40 cycles of 94 °C for 30 seconds, 55 °C for for 30 seconds, and 72 °C for 30 seconds; and a final step at 72 °C for 10 minutes.

Samples were then tested for the presence of *Wolbachia* using primers for *Wolbachia wsp* (forward primer 5′ AGT TGA TGG TAT TAC CTA TAA G 3′ reverse primer 5′ TGA CTT CCG GAG TTA CAT CAT AAC 3′, product size 410 bp) and *Wolbachia* GroEL (forward primer 5′ TTT GAT CGC GGT TAT C 3′ and reverse primer 5′ AGA TCT TCC ATC TTG ATT CC 3′, product size 410 bp) genes. Nuclease free water was used as negative control and female tsetse fly DNA (*Glossina morsitans morsitans*) from the lab line maintained at Yale University insectarium was used as a positive control. PCR reactions were performed using the following conditions: denaturation at 94 °C for 3 minutes, followed by 38 cycles consisting of at 94 °C for 30 seconds, at 50 °C for 30 seconds, and at 72 °C for 30 seconds; and a final extension for 10 minutes at 72 °C.

In addition, the DNA samples were analyzed for the presence of *Spiroplasma* utilizing primers against 16S rDNA (forward primer 5′ GGG TGA GTA ACA CGT ATC T 3′ and reverse primer 5′ CCT TCC TCT AGC TTA CAC TA 3′, product size 1000 bp). Nuclease free water was used as negative control and female tsetse fly DNA (*Glossina fuscipes fuscipes*) obtained from the field in Uganda was used as a positive control. PCR reactions were performed using the following conditions: denaturation at 94 °C for 3 minutes, followed 38 cycles consisting of at 94 °C for 30 seconds, at 55 °C for 30 seconds, and at 72 °C for 1.30 seconds; and a final extension for 10 minutes at 72 °C.

The resulting PCR products were analyzed by agarose gel-electrophoresis on a 1% gel, stained with ethidium bromide and recorded using the Gel Doc EQ quantification analysis software (Bio-Rad, Image Lab Software Version 4.1).

The amplified DNA fragments of the correct size were excised from the agarose gel and extracted using the Monarch DNA Gel Extraction Kit (New England Biolabs, Inc.). The purified PCR products were ligated into pGEM-T Vector (Promega, Madison, WI) and recombinant plasmids were used to transform competent *Escherichia coli* strain DH5α. Transformed cells were cultured and recombinant plasmid was extracted using the QIAprep Spin Miniprep Kit (Qiagen, Valencia CA). The purified DNA was sequenced in at the Yale Keck DNA Sequencing Facility.

## Electronic supplementary material

Supplementary Information

Supplementary Information (Original gel figures)

Sequence File
