# Supplementary material for: Investigation of Wolbachia spp. and Spiroplasma spp. in Phlebotomus species by molecular methods
Source: Sci Rep. 2018 Jul 13;8:10616. doi: 10.1038/s41598-018-29031-3 (PMC6045589; doi:10.1038/s41598-018-29031-3)
Supplement: Supplementary file 1 — Supplementary Information [file 41598_2018_29031_MOESM1_ESM.doc]

**Investigation of *Wolbachia spp*. and *Spiroplasma* spp. in *Phlebotomus* species by molecular methods**

Bilge KARATEPE^1^, Serap AKSOY^2^, Mustafa KARATEPE^1^

^1^ Niğde Ömer Halisdemir University, Bor Vocational School, Bor-Niğde, Turkey

^2^ Yale University, School of Public Health, Department of Epidemiology of Microbial Diseases, New Haven, Connecticut, United States of America

**Research Results**

Sandfly beta-tubulin was successfully amplified with standard PCR amplification conditions for all samples (Figure 1 shows the results from a subset of the samples).


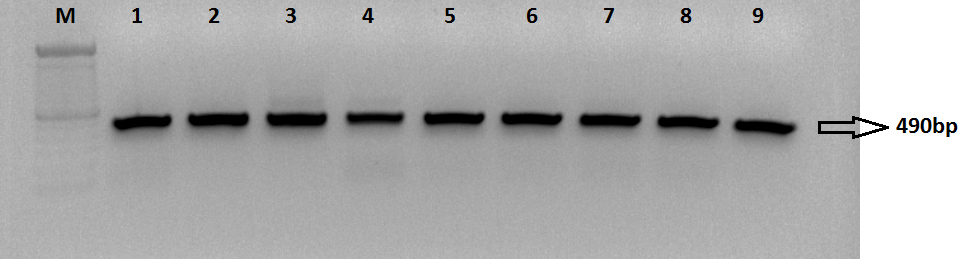


Figure 1. PCR products amplified with sandfly beta-tubulin specific primers.

M: DNA ladder (100bp), 1-9: Individual sandfly samples tested (490bp).

Detection of *Wolbachia* was accomplished using the *Wolbachia* *WSP* and *Groel* specific primer sets (Figure 2 and 3).


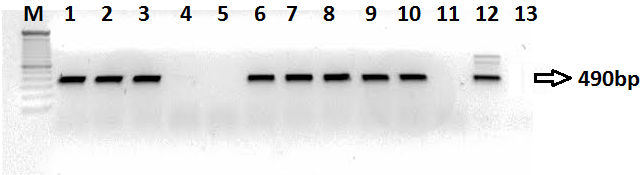


Figure 2: PCR products amplified with *Wolbachia* WSP specific primers. M: DNA ladder (100bp), 1,2,3,6-10: Positive samples, 4,5,11: Negative samples, 12: Positive control (490bp), 13: Negative control


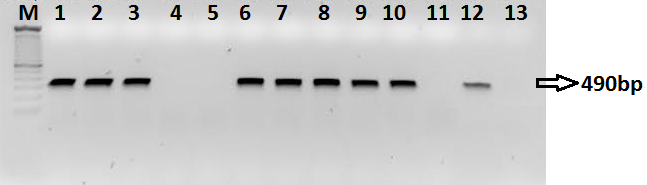


Figure 3: PCR products amplified with *Wolbachia* GroEl specific primers. M: DNA ladder (100bp), 1,2,3,6-10: Positive samples, 4,5,11: Negative samples, 12: Positive control (490bp), 13: Negative control


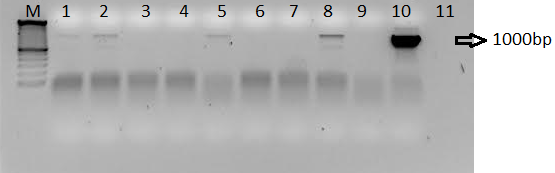


Figure 4: PCR products of amplified with *Spiroplasma* 16S specific primers. M: DNA ladder (100bp), 1,2,5,8: Positive samples, 3,4,6,7,9: Negative samples, 10: Positive control (1000bp), 9: Negative control

The amplified products were sequenced and the DNA sequence was analyzed by BLASTN analysis. The BLASTN results indicate that the sand fly *Wolbachia* *wsp* sequence shares the highest identity with the partial surface protein gene sequences (*wsp*) of *Wolbachia* endosymbiont of *Bactrocera neohumeralis* (100% identity: GenBank Accession Number KC668323) as well as the mosquito *Armigeres subalbatus* (100% identity: GenBank Accession Number KY523672). However, given the high conservation of this sequence, many different *Wolbachia* isolates showed 100% identity over the region analyzed. However, it appears that this strain of *Wolbachia* belongs to the Supergroup A. The sequences of three clones derived from 3 infected sand flies were identical.

The sequencing of the *groEl* gene of the clones was identical to the partial sequence derived from a *Wolbachia* endosymbiont of *Drosophila melanogaster* (100% identity: GenBank Accession Number AE017196) and *D. simulans* wHa (100% identity: GenBank Accession Number CP003884) based on BLASTN analysis. All clones had identical sequence. Given the high conservation of the *groEL* gene sequences, the sandfly *Wolbachia* sequence showed 100% identity with many other insect *Wolbachia* symbionts. However, many of the characterized *Wolbachia* strains that showed high similarity belonged to again Supergroup A.

In regards to *Spiroplasma* amplification products, the fragments were sequenced and subjected to BLAST analysis. The closest resulting match of the putative 16S rRNA fragments was to the 16S rRNA gene from Uncultured *Spiroplasma* sp. clone A9-24 (100% coverage, 100% identity: GenBank Accession Number KT983889.1).
