# Supplementary figures and images for: Investigation of Wolbachia spp. and Spiroplasma spp. in Phlebotomus species by molecular methods
Source: Sci Rep. 2018 Jul 13;8:10616. doi: 10.1038/s41598-018-29031-3 (PMC6045589; doi:10.1038/s41598-018-29031-3)

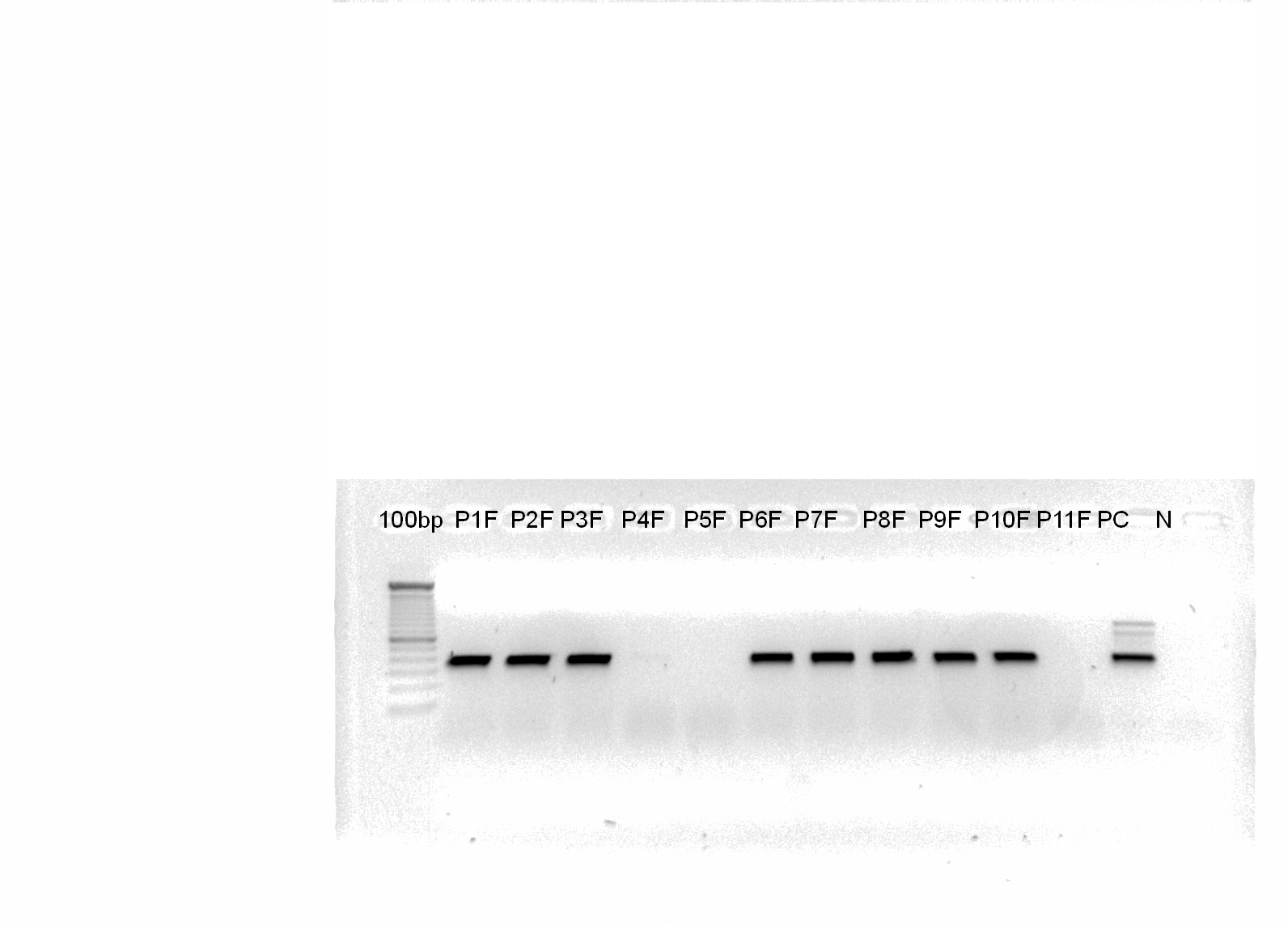

Supplement: Supplementary file 2 — Supplementary Information (Original gel figures) [file 41598_2018_29031_MOESM2_ESM.zip › Supplementary Information file (Original gel figures)/Figure 2. PCR products amplified with Wolbachia WSP specific primers.jpg]

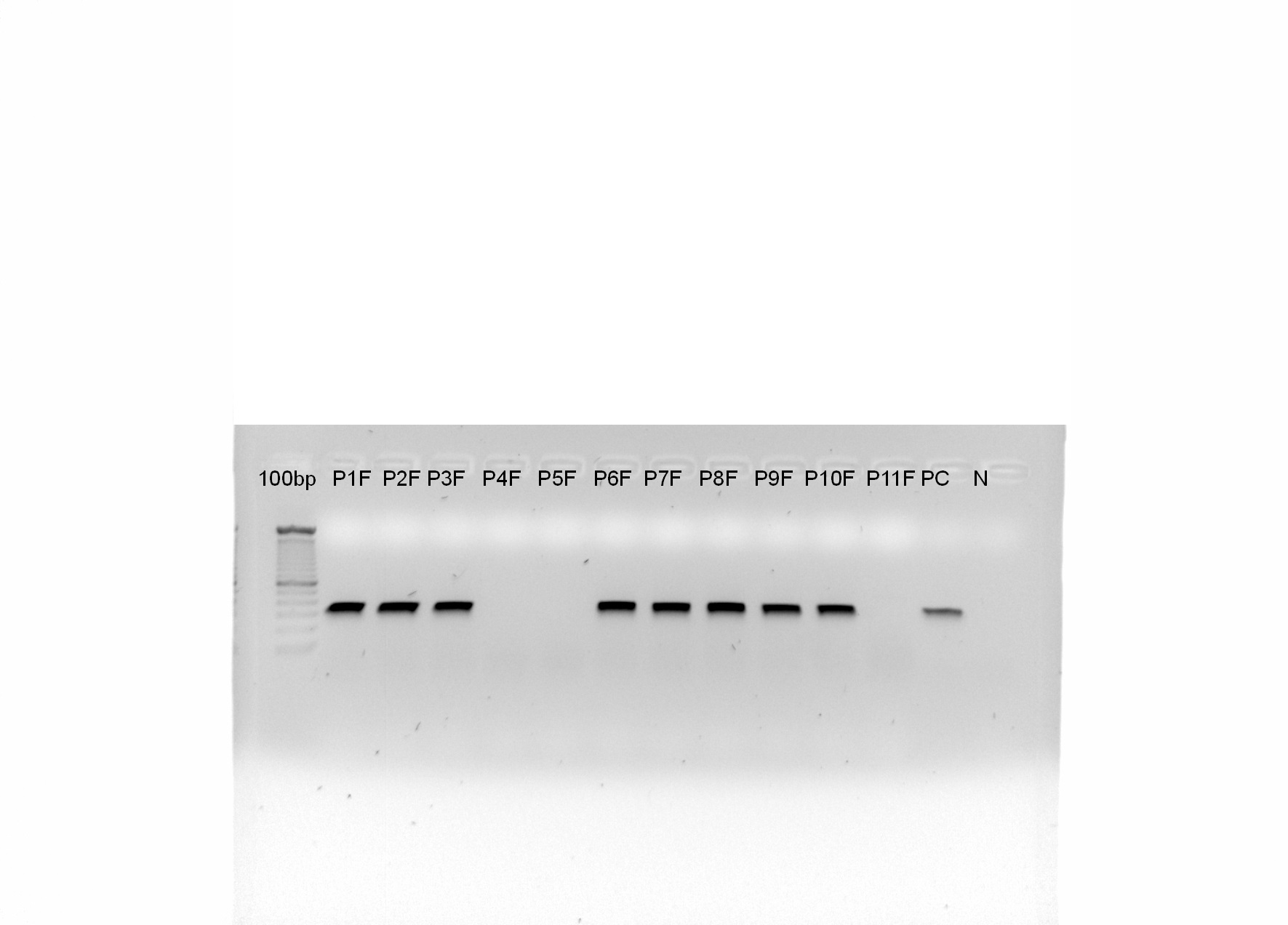

Supplement: Supplementary file 2 — Supplementary Information (Original gel figures) [file 41598_2018_29031_MOESM2_ESM.zip › Supplementary Information file (Original gel figures)/Figure 3. PCR products amplified with Wolbachia GroEl specific primers.jpg]

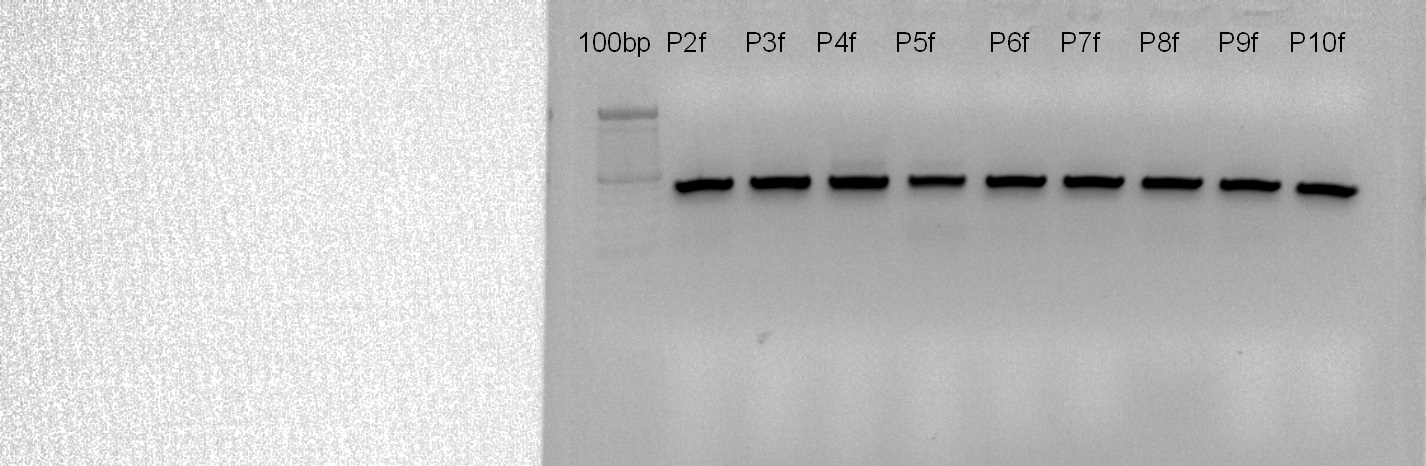

Supplement: Supplementary file 2 — Supplementary Information (Original gel figures) [file 41598_2018_29031_MOESM2_ESM.zip › Supplementary Information file (Original gel figures)/Figure 1. PCR products amplified with sandfly beta-tubulin specific primers..jpg]

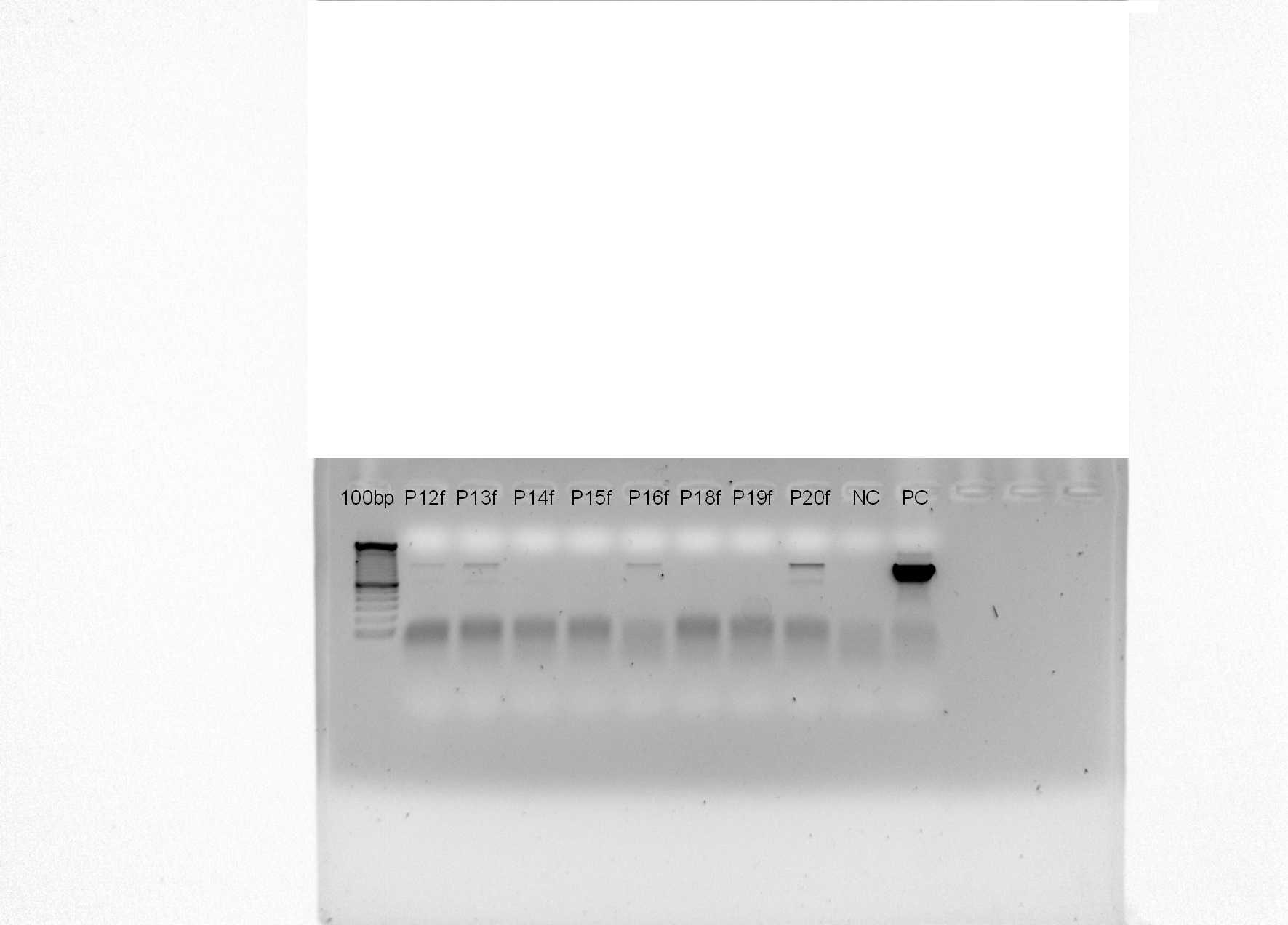

Supplement: Supplementary file 2 — Supplementary Information (Original gel figures) [file 41598_2018_29031_MOESM2_ESM.zip › Supplementary Information file (Original gel figures)/Figure 4. PCR products of amplified with Spiroplasma 16S specific primers.jpg]
